# Investigation of Chromosomal Structural Abnormalities in Patients With Undiagnosed Neurodevelopmental Disorders

**DOI:** 10.3389/fgene.2022.803088

**Published:** 2022-04-14

**Authors:** Ye Cao, Ho Ming Luk, Yanyan Zhang, Matthew Hoi Kin Chau, Shuwen Xue, Shirley S. W. Cheng, Albert Martin Li, Josephine S. C. Chong, Tak Yeung Leung, Zirui Dong, Kwong Wai Choy, Ivan Fai Man Lo

**Affiliations:** ^1^ Department of Paediatrics, The Chinese University of Hong Kong, Hong Kong SAR, China; ^2^ Department of Obstetrics and Gynaecology, The Chinese University of Hong Kong, Hong Kong SAR, China; ^3^ Key Laboratory for Regenerative Medicine, Ministry of Education (Shenzhen Base), Shenzhen Research Institute, The Chinese University of Hong Kong, Shenzhen, China; ^4^ Hong Kong Hub of Paediatric Excellence, The Chinese University of Hong Kong, Hong Kong SAR, China; ^5^ Clinical Genetic Service, Department of Health, Hong Kong SAR, China

**Keywords:** structural variations, mate-pair genome sequencing, neurodevelopmental disorders, Absence of heterozygosity (AOH), CNV (copy number variant), insertion, inversion, complex rearrangements

## Abstract

**Background:** Structural variations (SVs) are various types of the genomic rearrangements encompassing at least 50 nucleotides. These include unbalanced gains or losses of DNA segments (copy number changes, CNVs), balanced rearrangements (such as inversion or translocations), and complex combinations of several distinct rearrangements. SVs are known to play a significant role in contributing to human genomic disorders by disrupting the protein-coding genes or the interaction(s) with cis-regulatory elements. Recently, different types of genome sequencing-based tests have been introduced in detecting various types of SVs other than CNVs and regions with absence of heterozygosity (AOH) with clinical significance.

**Method:** In this study, we applied the mate-pair low pass (∼4X) genome sequencing with large DNA-insert (∼5 kb) in a cohort of 100 patients with neurodevelopmental disorders who did not receive informative results from a routine CNV investigation. Read-depth-based CNV analysis and chimeric-read-pairs analysis were used for CNV and SV analyses. The region of AOH was indicated by a simultaneous decrease in the rate of heterozygous SNVs and increase in the rate of homozygous SNVs.

**Results:** First, we reexamined the 25 previously reported CNVs among 24 cases in this cohort. The boundaries of these twenty-five CNVs including 15 duplications and 10 deletions detected were consistent with the ones indicated by the chimeric-read-pairs analysis, while the location and orientation were determined in 80% of duplications (12/15). Particularly, one duplication was involved in complex rearrangements. In addition, among all the 100 cases, 10% of them were detected with rare or complex SVs (>10 Kb), and 3% were with multiple AOH (≥5 Mb) locating in imprinting chromosomes identified. In particular, one patient with an overall value of 214.5 Mb of AOH identified on 13 autosomal chromosomes suspected parental consanguinity.

**Conclusion:** In this study, mate-pair low-pass GS resolved a significant proportion of CNVs with inconclusive significance, and detected additional SVs and regions of AOH in patients with undiagnostic neurodevelopmental disorders. This approach complements the first-tier CNV analysis for NDDs, not only by increasing the resolution of CNV detection but also by enhancing the characterization of SVs and the discovery of potential causative regions (or genes) contributory to could be complex in composition NDDs.

## Introduction

Structural variations (SVs), including various types of DNA changes (>50bps) in the genome, are known to contribute to the genomic diversity of the populations. Some of them are also associated with various genetic diseases (Abel et al., 2020; Ho et al., 2020). SVs can be balanced where there are no major gains or losses of genomic content but change(s) the organization of chromosomal segments, such as translocations, inversions, insertions; in unbalanced forms, commonly known as copy number variations (CNVs), or in complex forms with combination of several categories even involving multiple chromosomes. Medical studies or even presumably healthy human population genomic profiling studies reveal that simple SVs defined by conventional methods, such as karyotyping or chromosomal microarray analysis (CMA), could be complex in composition by next-generation sequencing studies (Dong et al., 2021). Current studies demonstrate that SVs are frequently seen, and balanced forms would be more likely seen in asymptomatic individuals, whereas complex rearrangements involving CNVs are also commonly identified in the human germline genome (de Pagter et al., 2015; Bertelsen et al., 2016; Collins et al., 2017). Rare SVs disrupting the coding sequences or interaction with regulatory elements, or adversely affecting the expressions of those disease-associated genes, are the known underlying mechanisms contributory to human diseases (Collins et al., 2017; Pocza et al., 2021). Therefore, reliable approaches to comprehensively and cost effectively identify clinically significant SVs in human genome, which is an important type of genetic variants and largely still underappreciated by current methods, are warranted.

Neurodevelopmental disorders (NDDs) are a group of disorders primarily associated with neurodevelopmental dysfunctions such as autism spectrum disorder (ASD), developmental delay (DD), and intellectual disability (ID). It is estimated that gene dosage alterations caused by large CNVs are responsible for 10–15% of NDD cases (Miller et al., 2010; Kaminsky et al., 2011; Yuan et al., 2021), while single-nucleotide variants (SNVs) and/or small insertions/deletions (InDels) contribute to over 30% of overall NDD cases (Srivastava et al., 2019). Despite extensive research and advancements in genetic diagnosis of neurological disorders, there are still at least half of NDD patients who remain idiopathic. In the last few decades, CMA has been recommended as the first-tier test for genetic investigation of NDDs (Miller et al., 2010), while currently, exome or genome sequencing (GS) is set as the second-tier testing (Manickam et al., 2021). However, these technologies mainly detect CNVs and SNVs/InDels, but are limited in identifying the direction/orientation of CNVs, let alone those balanced SVs. For example, CMA cannot determine whether a copy number gain is a forward tandem or reverse duplication, or an insertion resulting in inconclusive classification and interpretation. In addition, structural rearrangements cryptic to conventional G-banded chromosome analysis are largely known in NDDs. Apart from affecting the protein-coding portion of the genome, SVs can cause diseases by altering the copy number or position of regulatory elements, or by reshuffling higher-order chromatin structures as demonstrated in NDDs (D’haene and Vergult, 2021). For instance, the importance of translocations, inversions, and inversion-mediated complex structural rearrangements in autism spectrum disorder (ASD) and congenital anomalies have been demonstrated to be disease related by showing gene disruption or dysregulation due to a disruption of topologically associated domains (TADs) (Talkowski et al., 2012; Collins et al., 2017; Werling et al., 2018; Pocza et al., 2021). Last, some NDDs are caused by uniparental disomy (UPD) due to the involvement of imprinting genes, while some of them are caused by the homozygous defects in autosomal recessive genes due to parental consanguinity, both of which have one or more DNA stretches with the absence of heterozygosity (AOHs) identified in the genome (Fan et al., 2013; Palumbo et al., 2015; Yu et al., 2016).

Currently, increasing studies on the development of sequencing approaches and detection algorithms show the improvement of SV detection accuracy. Particularly, our previous studies have demonstrated our in-house mate-pair library construction and low-pass genome sequencing (>4-fold) enable comprehensive detection of structural rearrangements, cryptic to conventional karyotyping, as well as long contiguous regions of AOH contributed by UPD or parental consanguinity. Herein, we aim to (1) investigate the genomic composition of deletions and duplications with inconclusive significance identified by previous CNV analysis, and (2) characterize structural rearrangements and AOHs (likely resulted from UPD or parental consanguinity) by utilizing mate-pair genome sequencing in 100 NDD cases.

## Materials and Methods

### Subjects

The study was approved by the Joint Chinese University of Hong Kong—New Territories East Cluster Clinical Research Ethics Committee (CREC Ref. No. 2019.600). DNA samples of 100 consecutive patients were retrieved for this study. These patients (1) were referred to Clinical Genetic Service, Department of Health, Hong Kong SAR during 2019–2020; (2) have major indications including developmental delay, intellectual disability, congenital abnormalities, and autism spectrum disorders; (3) with a negative or inconclusive finding from previous CNV analysis (at a resolution of 50 kb for all types of CNVs; for homozygous or hemizygous deletions, the resolution was set as 10 kb due to the absence of aligned reads) by low-pass GS (a minimal of 15 million reads) as we described previously (Wang et al., 2020). Inconclusive findings included CNVs classified as a variant of uncertain significance, such as intragenic duplications or deletions involving an autosomal recessive gene.

### Mate-Pair Genome Sequencing

2 µg of genomic DNA from each case was sheared to fragment sizes ranging from 3 to 8 kb with a red mini-tube on a Covaris device (Covaris, Inc., MA, United States). The fragmented DNA was then prepared for mate-pair library construction following our reported protocols (Dong et al., 2019b). The libraries were sequenced on an MGISEQ-2000 platform (MGI Tech Co., Ltd., Shenzhen, China) for a minimum of 60 million read pairs (paired-end 100 bp) per sample, equivalent to ∼ 4X sequencing read-depth.

### Genomic Variant Detection

After data QC, the read-pairs were aligned to the human reference genome (GRCh37/hg19) using the Burrows–Wheeler aligner (BWA)(Li and Durbin, 2009). CNV, structural rearrangement (or structural variant, SV), and absence of heterozygosity (AOH) detection were performed according to our previously reported methods (Dong et al., 2019a; Wang et al., 2020; Dong et al., 2021).

CNV detection: Uniquely aligned reads were classified into both adjustable sliding windows (50 kb with 5 kb increments) and non-overlapping windows (5 kb), independently. Subsequently, the copy ratios of all windows were normalized by GC% and our in-house population-based dataset (Chau et al., 2020; Wang et al., 2020). Region(s) with CNV were detected, and the precise boundaries of each CNV were identified by an increment-rate-of-coverage module (Dong et al., 2016) at a resolution of 50 kb. For homozygous or hemizygous deletions, it was reported if there were more than one non-overlapping window with an extremely low number of aligned reads (0.1 as copy ratio) or even absence of aligned read (copy ratio equaled to 0). The minimal size of a reported homozygous or hemizygous deletion was approximately 10 kb.

SV identification: Chimeric read-pairs defined as read-pairs aligned to different chromosomes or to the same chromosome with a genomic distance>=10 kb were selected for event clustering. Each potential event was then filtered against a dataset of systematic errors as well as with optimized parameters (such as minimal of read-pairs supported and the orientation of aligned read-pairs) as described in our previous studies (Dong et al., 2014; Dong et al., 2019b).

AOH analysis: Reads due to PCR duplication were removed, and the coverage of each genomic location was summarized by using the Mpileup module from SAMtools. A genomic locus with a read-depth of 5- to 20-fold with read(s) covered and with at least one read supporting a mutant base type was selected for the determination of heterozygous or homozygous SNV. The number of heterozygous SNVs and homozygous SNVs were calculated per window (with fixed size: 100-kb), respectively, and normalized by the average rate in that sample. Regions with AOH were indicated by a simultaneous decrease in the rate of heterozygous SNVs and increase in the rate of homozygous SNVs (Dong et al., 2021).

Candidate CNVs, SVs, and AOHs were filtered against our in-house datasets, the 1,000 Genomes Project, and gnomAD SVs to filter the known common variants in the populations.

### Variant Verification

For verification of structural rearrangements, rearrangement junction-specific PCR and Sanger sequencing were performed (Dong et al., 2014). Primers were designed by using the online software Primer3, Primer-Blast (NCBI), and *in silico* PCR (UCSC). PCR was performed in case and negative control simultaneously, and the products were sequenced on an ABI 3730 DNA Analyzer (Applied Biosystems, Foster City, CA, United States). The Sanger sequencing results were aligned to the reference genome by BLAT (UCSC) for breakpoint verification and delineation.

For CNV verification, qPCR with primers targeting the candidate region was performed as previously described (Wang et al., 2020). Primers were designed with Primer 3 Web, Primer-Blast (NCBI), or *in silico* PCR (UCSC) based on the reference genome (GRCh37/hg19). The melting curve analysis was carried out for each pair of primers to ensure specificity of the PCR amplification, and the standard curve method was used to determine PCR efficiency (within a range of 95 – 105%). Each reaction was performed in duplicate in 10-μL of reaction mixtures simultaneously in case and control (in-house normal male and female controls) using the SYBR Select Master Mix (Applied Biosystems). The reactions were run on a 7900HT Real-Time PCR System (Applied Biosystems) using the default reaction conditions. The copy numbers in each sample were determined by the ΔΔ Ct (cycle threshold) method, which compared the difference in Ct of the targeted region with a reference primer pair targeting a universally conserved element in a case against control.

For verification of AOH, a well-established, customized CMA 8X60k Fetal DNA Chip v2.0 (Agilent Technologies, Santa Clara, CA, United States), containing both SNP and comparative genomic hybridization (CGH) probes, was used as previously described (Chau et al., 2019). CNV and AOH analyses were evaluated with CytoGenomics (Agilent).

### Annotation and Pathogenicity Prediction

For CNVs and SVs, the breakpoints/boundaries identified by mate-pair GS were used for annotation: (1) direct disruption or involvement of gene(s), or (2) disruption of topologically associated domains (https://www.clintad.com/single/) in which with gene(s) involved. For CNV/SV potentially involving gene(s) that was an OMIM disease-causing gene, or a disease-causing gene due to haploinsufficient/triplosensitivity in peer-reviewed publications, or by ClinGen Dosage Sensitivity Map (https://dosage.clinicalgenome.org/), DECIPHER (https://www.deciphergenomics.org/), or gnomAD (https://gnomad.broadinstitute.org/), it was subjected for further analysis.

For AOHs, if there were multiple regions with AOH (>5 Mb) reported in a case, the overall size was calculated as the sum of all regions with AOHs excluding the ones in sex chromosomes. In contrast, if there were more than one region of AOH identified in one chromosome, uniparental disomy was suspected when the size of interstitial AOH exceeded 15 Mb or the size of terminal AOH exceeded 5 Mb based on the ACMG guideline (Del Gaudio et al., 2020).

## Results

### Cohort Summary and Mate-Pair Genome Sequencing

In this study, 100 patients (71 male and 29 female) were recruited from 2020 to 2021. All participants were examined by clinical geneticists and received a negative result (*n* = 76) or an inconclusive finding (*n* = 24) by previous sequencing-based CNV analysis (Table 1). This cohort presented a spectrum of clinical features, mainly involving neurodevelopmental conditions such as intellectual disability and ASD, with or without comorbidities such as dysmorphology, seizure, and hypotonia. Among them, 13 cases (13%) had other congenital anomalies or organ-specific dysfunction (Supplementary Table S1).

**TABLE 1:** Detection results of two methods of the 24 inconclusive cases.

Case ID	Clinical details	Fetalseq CNV results	Reported results	Mate-pair genome sequencing results
**Deletion**				
1	Delay	seq[GRCh37] del(5)(q14.3) chr5:g.90028949_ 90237360del	Pathogenic variant on autosomal recessive gene	seq[GRCh37] del(5)(q14.3) chr5:g.90027969_90240857del
8	Delay	seq[GRCh37] del(2)(p24.1) chr2:g.20082407_20142043del	Pathogenic variant on autosomal recessive gene	seq[GRCh37] del(2)(p24.1) chr2:g.20080939-20139774del
10	Delay	seq[GRCh37] del(6)(q12) chr6:g.65418244_65760319del	Pathogenic variant on autosomal recessive gene	seq[GRCh37] del(6)(q12) chr6:g.65415408-65763210del
21	Delay	seq[GRCh37] del(7)(q32.3q33) chr7:g.132543248_132639078del	VUS	seq[GRCh37] del(7)(q32.3q33) chr7:g.132542905-132639717del
26	Developmental delay and microcephaly	seq[GRCh37] del(12)(p11.23) chr12:g.26992893_27345229del	VUS	seq[GRCh37] del(12)(p11.23) chr12:g.26991317-27342205del
37	Bilateral severe hypoplastic vestibular nerve and global delay, ADHD	seq[GRCh37] del(22)(q11.22) chr22:g.22313025_22572225del	VUS	seq[GRCh37] del(22)(q11.22) chr22:g.22313363_22579931del
38	Delay	seq[GRCh37] del(4)(q25) chr4:g.112915276_113354558del	VUS	seq[GRCh37] del(4)(q25) chr4:g.112915198-113354258del
67	ASD, global delay	seq[GRCh37] del(8)(p21.3) chr8:g.19352596_19553354del	Pathogenic variant on autosomal recessive gene	seq[GRCh37] del(8)(p21.3) chr8:g.19352895-19553738del
71	Developmental delay	seq[GRCh37] del(9)(p24.3) chr9:g.99746_402497del	VUS	seq[GRCh37] del(9)(p24.3) chr9:g.110928_398513del
80	Autism, delay	seq[GRCh37] del(11)(p15.4) chr11:g.6907077_7058427del	VUS	seq[GRCh37] del(11)(p15.4) chr11:g.6910893_7062143del
**Duplication**				
4	Delay	seq[GRCh37] dup(8)(p23.2) chr8:g.3700597_5946301dup	VUS	dup(8)(8p23.2)(pter->8p23.2(+)(5951139)::q21.3(+)(3686605)- > qter)
9	Epilepsy with mild delay	seq[GRCh37] dup(13)(q13.3) chr13:g.37265048_37433772dup	VUS	dup(13)(q13.3)(pter- > q13.3(+)(37430811)::q13.3(+)(37267951)- > qter)
13	Delay	seq[GRCh37] dup(13)(q12.3q13.2) chr13:g.30805367_34307738dup	VUS	dup(13)(q12.3q13.2)(pter- > q13.2(+)(34291095)::q12.3(+)(30797601)- > qter)
17	Delay	seq[GRCh37] dup(11)(p15.4) chr11:g.9533650_10145145dup	VUS	dup(11)(p15.4)(pter- > p15.4(+)(10148395)::p15.4(+)(9533106)- > qter)
19	Autism, developmental delay	seq[GRCh37] dup(17)(p13.1) chr17:g.6989477_7347779dup	VUS	Complex rearrangement
27	Delay	seq[GRCh37] dup(3)(q25.32) chr3:g.158051611_158591897dup	VUS	dup(3)(q25.32)(pter- > q25.32(+)(158590381)::q25.32(+)(158051006)- > qter)
29	Bilateral congenital hearing loss, history of delay	seq[GRCh37] dup(10)(q22.2) chr10:g.76002141_76107403dup	Pathogenic variant on autosomal recessive gene	dup(10)(q22.2)(pter- > q22.2(+)(76114070)::q22.2(+)(76001841)- > qter)
36	Delay	seq[GRCh37] dup(15)(q21.3) chr15:g.54467876_55401968dup	VUS	dup(15)(q21.3)(pter- > q21.3(+)(55445120)::q21.3(+)(54466811)- > qter)
40	Delay	seq[GRCh37] dup(22)(q11.23) chr22:g.23674079_25063169dup	VUS	LCR
48	Delay FTT, left corneal opacity, dysmorphism	seq[GRCh37] dup(6)(p12.3) chr6:g.46876528_47353335dup	VUS	dup(6)(p12.3)(pter- > p12.3(+)(47364590)::p12.3(+)(46875330)- > qter)
49	Delay	seq[GRCh37] dup(8)(p23.1) chr8:g.8093423_9166490dup	VUS	LCR
55	Delay, subtle dysmorphism	seq[GRCh37] dup(7)(q11.22) chr7:g.69820533_70172074dup	VUS	dup(7)(q11.22)(pter- > q11.22(+)(70166997)::q11.22(+)(69827447)- > qter)
56	Delay	seq[GRCh37] dup(4)(q32.3) chr4:g.165050961_165626257dup	VUS	dup(4)(q32.3)(pter- > q32.3(+)(165626043)::q32.3(+)(165052397)- > qter)
65	Autism, developmental delay	seq[GRCh37] dup(7)(q21.11) chr7:g.82027618_82168623dup	VUS	dup(7)(q21.11)(pter- > q21.11(+)(82155471)::q21.11(+)(82025319)- > qter)
80	Autism, delay	seq[GRCh37] dup(3)(p12.3) chr3:g.79128426_79237810dup	VUS	dup(3)(p12.3)(pter- > p12.3(+)(79237826)::p12.3(+)(79128870)- > qter)

### Investigation of Inconclusive CNVs Reported in Previous Analysis

Among them, 24 cases were referred due to the inconclusive results of CNV analysis which cannot fully explain patients’ phenotype, including 15 duplications and 10 deletions. In one case, patient 80 had two CNVs, including a deletion and duplication. We aimed to validate the consistency of CNV detection, and to investigate the directions/orientations of the duplications. We employed both read-depth-based and chimeric-read-pair-based algorithms for CNV detection.

Twenty-five CNVs reported in 24 cases were all detected by mate-pair GS. We also compared the locations of boundaries for the CNVs reported by each method as mate-pair GS enabled the identification of chimeric read-pairs to narrow down the candidate regions of CNV/SVs’s breakpoint junctions. These two approaches yielded similar sizes of these 25 CNVs. For ten deletions, the minor discrepancies in the breakpoint coordinates did not affect the clinical interpretation of the CNVs (Table 1).

Among the 15 duplications with inconclusive findings, we aimed to determine the directions/orientations of these duplication segments (i.e., tandem forward or reverse duplications, insertions, or complex rearrangements) by chimeric read-pairs. Among them, 12 were identified as forward tandem duplications, and one was found to be involved in complex rearrangements (patient 19). However, the genomic compositions of the other two duplications were unable to be identified by mate-pair GS due to the presence of segmental duplications flanking the CNVs of these two regions: 22q11.23 (patient 40) and 8p23.1 (patient 49) (Table 1).

### Additional CNV and SV Findings Among all 100 Cases

By using chimeric read-pair analysis among all 100 cases, mate-pair GS revealed five cryptic deletions from four cases, with a size ranging from 8.5 to 46 kb, and ten rare SVs detected from 10 cases including five balanced inversions, and one simple and four complex insertions (Table 2). Patient 15 was detected with an 8.5 kb heterozygous deletion involving exon 1 of the *ASAH1* gene which is known to be associated with autosomal recessive spinal muscular atrophy with progressive myoclonic epilepsy [MIM159950]. The deletion was classified as pathogenic CNV in an autosomal recessive gene and confirmed by qPCR (Supplementary Figure S2). Patient 23 was detected with 9.5 kb heterozygous deletion involving three exons of the *ANKRD26* gene, which is associated with autosomal dominant thrombocytopenia [MIM 18800] (Table2). It indicated a further hematological test was warranted in this patient; however, there was not enough gDNA left for validation. This deletion was further classified as VUS.

**TABLE 2:** List of additional SVs detected in this cohort.

Case ID	FetalSeq (CNV analysis)	Additional findings	Size (bps)	Gene(s) on breakpoints
**Deletion**				
42	Negative	seq[GRCh37] del(9)(q21.32) chr9:g.85918802_85929907del	11,105	*FRMD3*
15	Negative	seq[GRCh37] del(8)(p22) chr8:g.17937910_17946394del;	8,484;	*ASAH1*
		seq[GRCh37] del(1)(q21.3) chr1:g.152250046_152295889del	45,843;	*FLG*
21	VUS, seq[GRCh37] del(7)(q32.3q33) chr7:g.132543248_132639078del	seq[GRCh37] del(17)(q25.1) chr17:g.70909687_70947878del	38,191	*SLC39A11*
23	Negative	seq[GRCh37] del(10)(p12.1) chr10:g.27294954_27304416del	9,462	*ANKRD26*
**Inversion**				
4	VUS, seq[GRCh37] dup(8)(p23.2) chr8:g.3700597_5946301dup	seq[GRCh37] inv(14)(q21.2)(pter- > q21.2(+)(44888815)::q21.2(-)(44950538)<-q21.2(-)(44890455)::q21.2(+)(44958120)- > qter)	69,305	—
25	Negative	seq[GRCh37] inv(1)(p22.3)(pter- > p22.3(+)(85672144)::p22.3(-)(85684901)<-p22.3(-)(85672336)::p22.3(+)(85685338)- > qter)	13,194	—
47	Negative	seq[GRCh37] inv(3)(p24.1)(pter- > p24.1(+)(94294177)::p24.1(-)(94319877)<-p24.1(-)(94296491)- > p24.1(+)(94320566)- > qter)	26,389	—
65	VUS, seq[GRCh37] dup(7)(q21.11) chr7:g.82027618_82168623dup	seq[GRCh37] inv(6)(q12)(pter- > q12(+)(66827535)::q12(-)(68075879)<-q12(-)(66828312)::q12(+)(68076174)- > qter)	1,248,639	—
66	Negative	seq[GRCh37] inv(8)(p11.1q11.1)(pter- > p11.1(+)(43669974)::q11.1(-)(48070098)<-p11.1(-)(43671748)::q11.1(+)(48071062)- > qter)inv(15)(q26.3)(pter- > q26.3(+)(100271705)::q26.3(-)(100487648)<-q26.3(-)(100272211)::q26.3(+)(100489231)- > qter)	4,401,088;	—
			217,526	
**Insertion**				
11	Negative	seq[GRCh37] ins(5;5)(q35.3;q35.3)(pter- > q35.3(+)(180499168)::q35.3(-)(180478893)<-q35.3(+)(180416486)::q35.3(+)(180501005)- > qter) dup(5)(q35.3) chr5:g.180416486_180478893dup	20,275	*BTNL3-BTNL9*
30	Negative	Dup ins and flanking dup		
		seq[GRCh37] ins(8;8)(p23.1;p23.3)(pter- > p23.1(+)(6513172)::p23.3(-)(1543512)<-p23.3(-)(1114809)::p23.1(+)(6439080)- > pter)	428,703;	*DLGAP2;*
		dup(8)(p23.3) chr8:g.1114809_1543512dup	74, 039	*MCPH11*
		dup(8)(p23.1) chr8:g.6439080_6513172dup		
36	VUS, seq[GRCh37] dup(15)(q21.3) chr15:g.54467876_55401968dup	Dup ins and flanking dup		
		seq[GRCh37] ins(8;8)(q23.1;q22.3)(pter- > q23.1(+)(1,10119574)::q22.3(-)(104589153)<-q22.3(-)(104465936)::q23.1(+)(109821483)- > qter) dup(8)(q22.3) chr8:g.104465936_104589153dup dup(8)(q23.1) chr8:g.109821483_1,10119574dup	123,217;	*RIMS2;*
			298,091	*TRHR*
45	Negative	Dup ins and flanking dup	42,546	*FMLN2, PRPF40A;*
		seq[GRCh37] ins(2;2)(q23.3;q23.3)(pter- > q23.3(+)(153563012)::q23.3(-)(153536242)<-q23.3(-)(153493696)::q23.3(+)(153542212)- > qter) dup(2)(q23.3) chr2:g.153493696_153536242dup		
		dup(2)(q23.3) chr2:g.153542212_153563012dup		*PRPF40A*
69	Negative	Unresolved complex rearrangement		—

### AOH Findings

**FIGURE 1:**
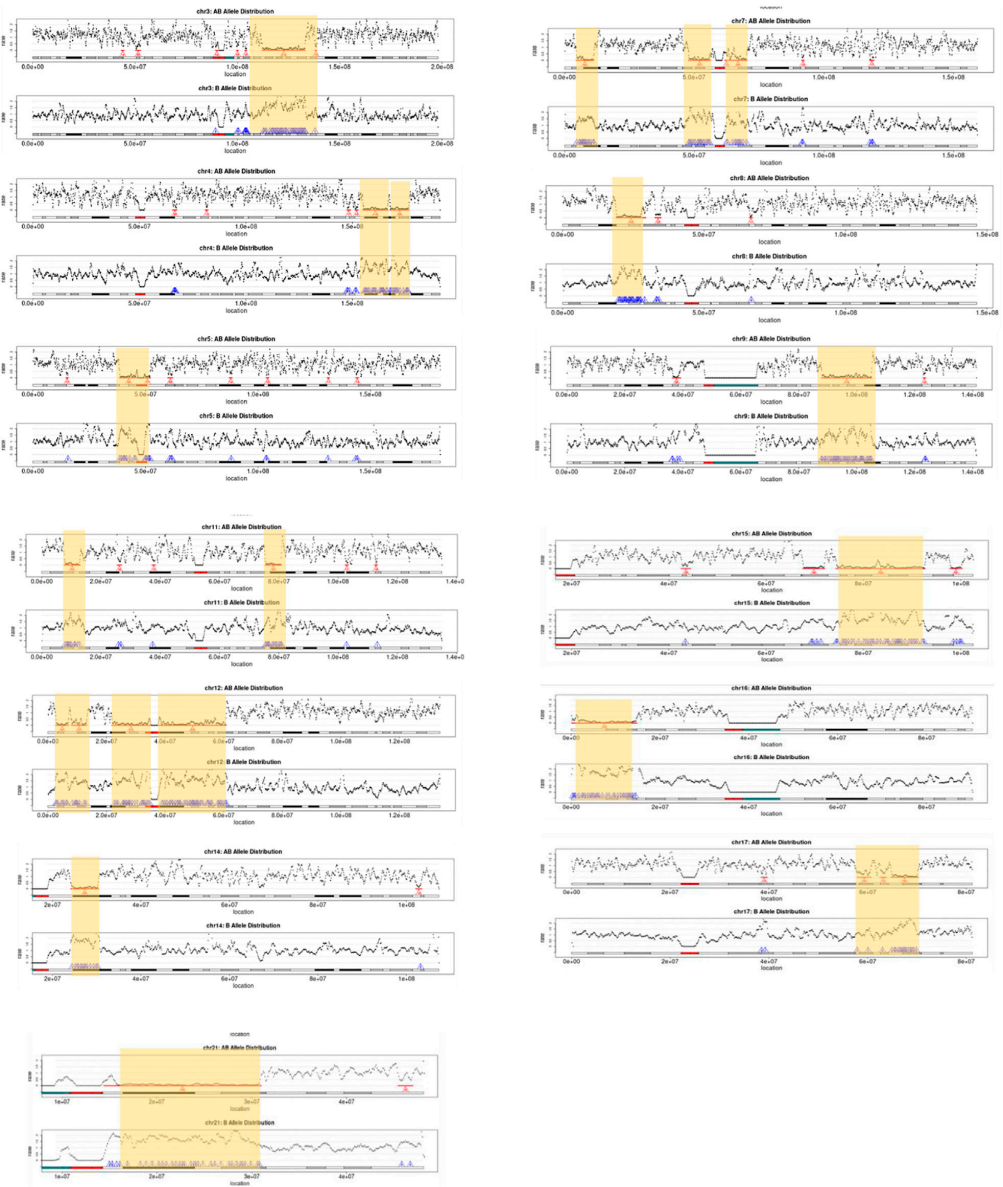
Regions of AOH detected in thirteen chromosomes of case 76. For each chromosome, the AOH regions detected are indicated by yellow highlighted boxes and red arrows, and the number of windows that support the AOH is shown in red (upper figure in each chromosome: AB allele distribution), while windows with an increased rate of homozygous SNVs within regions reported (lower figures in each chromosome: B allele distribution) are shown by blue arrows.

The absence of heterozygosity analysis was applied to each case (n = 100) to detect constitutional and mosaic AOH with a size at 5 Mb. Three cases (3%) were detected with multiple regions with AOH (≥ 5 Mb) identified including case 41 involving imprinting chromosomes (Supplementary Table S2). In case 76, a three-year-old boy with autism and delay received a negative result from previous CNV analysis. However, mate-pair GS identified multiple regions with AOH, the overall size of which summed to be 214.5 Mb involving 13 autosomes (Figure 1). Multiple regions with AOH in this case were verified by our CMA arrays (aCGH + SNP probes). Therefore, a familial relationship between the parents was suggested. However, this patient was lost to follow-up.

## Discussion

Our study showed the feasibility and advantages of applying mate-pair low pass GS in a cohort of 100 patients with neurodevelopmental disorders, and congenital abnormalities with inconclusive or negative findings from previous CNV analysis. Our work also demonstrated that mate-pair GS with a large DNA insert size (∼5 kb) and a minimal read-depth of 4-fold enables identification of DNA changes (CNVs or SVs) cryptic to previous CNV analysis, and delineation of the breakpoint junctions. Meanwhile, it also showed the robustness of AOH detection by utilizing such limited sequencing read-depth (4-fold).

Through chimeric-read-pair-based algorithm, we further confirmed the robustness of identifying the CNV boundaries by using a read-depth-based algorithm in previous CNV analysis with 0.25-fold genome sequencing data. It is consistent with our previous finding showing no significant differences of the CNV boundaries detected between two methods (Dong et al., 2016). However, the mate-pair-based algorithm shows its advantages in the following aspects.

First, it provides the genomic compositions of duplications with an inconclusive clinical significance: we re-evaluated the 15 duplications classified as VUS in this cohort detected by the previous CNV test (Table 1). Among these, the majority of duplications (80%, 12/15) were forward tandem duplication, the incidence of which is comparable to that previously reported (Newman et al., 2015). This suggests that genes located on the breakpoints in ∼80% of duplications would be intact. In contrast, if genes located on the breakpoints of duplications cause diseases that explain the phenotypes of the patients, it would highly warrant further evaluation of the orientation of the copy number gains. Still, we have two (13%) that cannot be identified due to flanking low copy repeats, which imply the long LCR would impact the SV detection by this mate-pair genome sequencing.

Second, it identifies small CNVs (<50 kb) that go beyond the resolution of testing through 0.25-fold GS. Five cryptic exonic deletions involving single genes were identified in this study. Although two small clinically significant deletions did not fully explain the patients’ neurological issue (case 15 and case 23), the accuracy of detecting such small CNVs have been confirmed by qPCR. In addition, exonic CNVs related to autosomal recessive disorders are often small in size and underappreciated due to the limitations in the routine CNV detection method such as CMA (Yuan et al., 2020). Therefore, mate-pair GS might increase diagnostic yield in cases contributed by small CNVs although this might not be a common cause in NDD patients in this study.

Third, it detects additional SVs and reveals complex rearrangements. In this cohort, five rare inversions were detected in five cases (5%), all of which were small paracentric inversions. But none of these inversions disrupted genes or their interactions with known regulatory elements. They were classified as VUS considering their rarity in the population. In addition, five insertions (5%) were detected, four out of which were involved in complex rearrangements. These five insertions were not related to those previously reported CNVs in each case. Interestingly, three of these four complex insertions were delineated as insertion (duplicated segments) with flanking duplications identified in the insertion site (Table 2). Although no gene disruption was observed, we still classify them as VUS. Genome-wide structure rearrangement discovery is challenging while increasingly attracting our attention with the improvement of sequencing detection methods. However, limited information about polymorphic SVs in the human genome hampers its clinical significance interpretation. Genes interrupted by the breakpoint seen in the patients would be current focus to correlate the diseases for interpretation.

**FIGURE 2:**
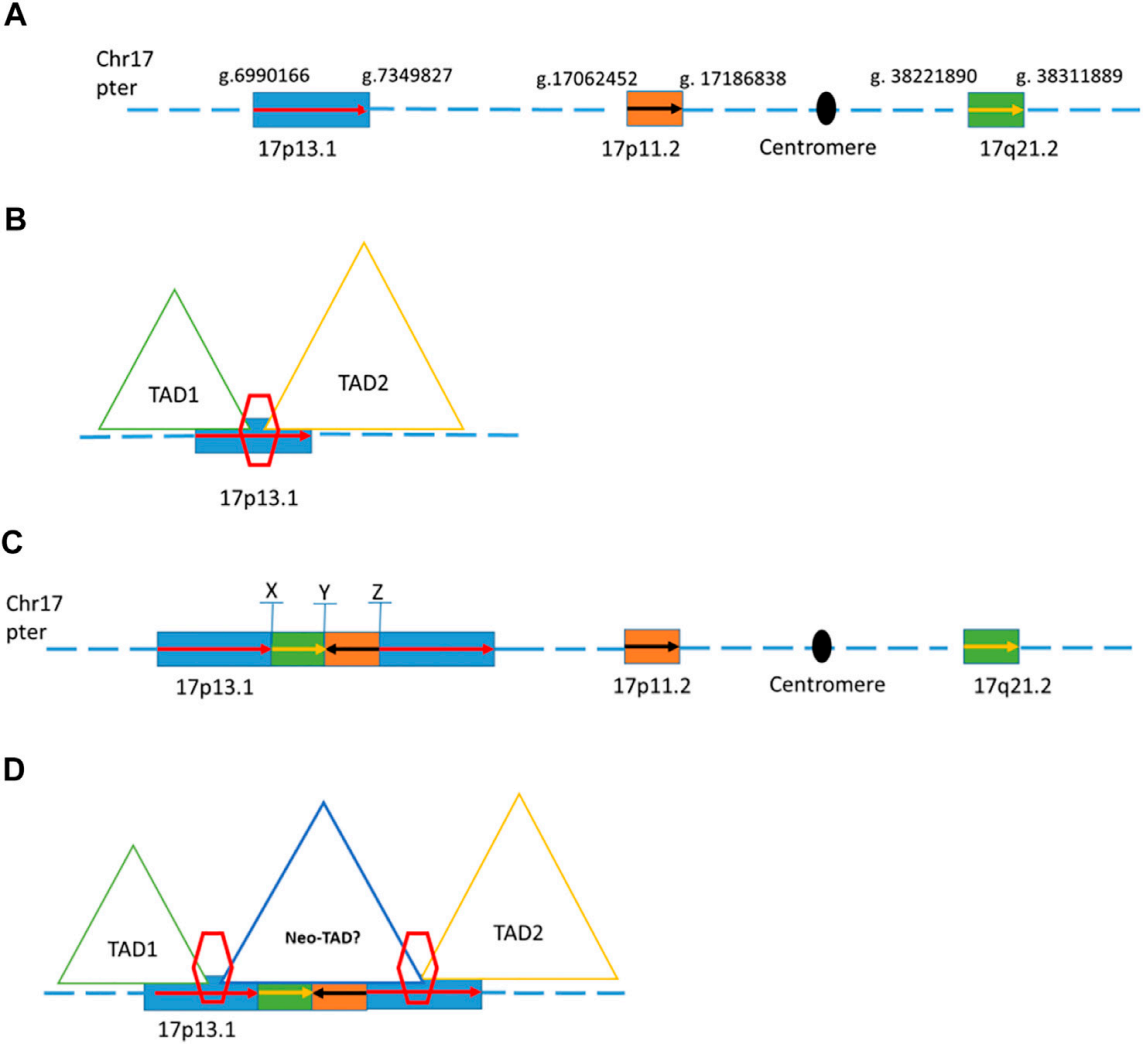
Schematic of genome structures in case 19. **(A)** Wild type of chromosome 17 with blocks of region that involved the complex rearrangement. **(B)** Two different topologically associating domains with a boundary on the 17p13.1 **(C)**. Schematic representation of one possible complex rearrangement on 17p13.1 involving duplications and insertions from 17p13.1, 17p11.2, and 17q21.2. X, Y, and Z indicate the breakpoints within this rearrangement. **(D)** Duplications of the boundary and the flanking regions (inter-TAD duplication) were proposed to change the overall chromatin architecture of the locus, creating a new chromatin domain (neo-TAD) on this complex rearrangement region.

One of the previously reported inconclusive CNVs was found to involve complex rearrangements based on the mate-pair GS result. Patient 19 was a five-year-old male child who showed autism, global developmental delay, and mild dysmorphic feathers. A *de novo* 358 kb duplication in chromosome 17 (seq[GRCh37] dup(17)(p13.1)dn chr17:g.6989477_7347779dup) was reported in a previous CNV analysis. Mate-pair GS detected another two genomic segments from distal location of chromosome 17 (a segment of 124 kb from 17p11.2 and a segment of 90 Kb from 17q21.2) inserted in the middle of these two copies of 358 kb segment of 17p13.1 (Figure 2). The composition of this complex rearrangement is shown in Figure 2C. The 124 kb insertion from 17p11.2 was in a reverse orientation. The three breakpoints were all validated by Sanger sequencing (Supplementary Figure S1). This 358 kb duplication is overlapped with a dosage-sensitive region on the 17p13.1 commonly leading to intellectual disability and microcephaly (Carvalho et al., 2014). Multiple patients with overlapping deletions or triplication changes shared microcephaly and intellectual disability, and defined the smallest region of overlapping (SRO) on the 17p13.1 as around 156 kb in size (GRCh37/hg19, chr17:g.7055654_7212104) (Carvalho et al., 2014). In addition, defects in the *DLG4* gene of this region are known to cause intellectual developmental disorder 62 (MIM 618793) due to haploinsufficiency (Lelieveld et al., 2016; Moutton et al., 2018). Mooneyham et al. reported two patients with neurodevelopmental delays and absolute/relative macrocephaly with a shared region of 62.5 kb on the 17p13.1, suggesting that *DULLARD*, *DLG4*, and *GABARAP* genes would be the candidate genes for neurodevelopmental delays identified in this patient (GRCh37/hg19, chr17:g.7094072_7156584) (Mooneyham et al., 2014). Currently, this 358 kb duplication is known to involve a TAD boundary (Figure 2B). The two insertions might result in an overexpression of those genes locating in the 358 kb duplication by bringing in additional regulatory elements to possibly promote certain ectopic enhancer-promoter interactions in the neo-TAD or expression of genes in the inserted regions (Figure 2D).

Last, it would identify regions with AOH. Small regions with AOH (<3 Mb) in the human genome are commonly seen, while regions with AOH are also known to cause diseases by unmasking of autosomal recessive allele or imprinting region. The prevalence of UPD associated with a clinical presentation due to imprinting disorders or recessive diseases ranges from 1 in 3,500 to 1 in 5,000 (Del Gaudio et al., 2020). Studies suggest reporting terminal long continuous stretches of homozygosity (LSCH) on each chromosome at a resolution of 5 Mb and interstitial LSCH at 15–20 Mb (Hoppman et al., 2018). In this study, we applied 5 Mb as the resolution for identifying regions with AOH as demonstrated in our previous publication (Dong et al., 2021). The result showed that 3% of cases from our cohort were reported to have regions with AOH on various chromosomes more than imprinting chromosome. One of the patients was detected with multiple AOHs in 13 chromosomes, with an overall size of 214.5 Mb. These large regions of homozygosity involving multiple chromosomes indicate a consanguineous relationship between the proband’s parents which was suggested to report as incidental findings based on the current laboratory’s reporting policy. Such information is also important for clinicians to further evaluate the possibility of any gene locating in regions of AOH is known to be associated with a patient’s presentation (Del Gaudio et al., 2020) as parental consanguinity is known to contribute to developmental delay or autism spectrum disorder due to the increased risks of autosomal recessive disorders.

In summary, this study showed the feasibility of mate-pair low-pass GS in patients with neurodevelopmental disorders who received negative or inclusive results from previous CNV analysis. This approach complements the first-tier CNV analysis for NNDs through not only increasing the resolution of CNVs detection but also better identification and delineation of chromosomal structural rearrangements as well as the discovery of potential causative regions (or genes) involved in regions with AOH.

## Data Availability

The datasets presented in this study can be found in online repositories. The names of the repository/repositories and accession number(s) can be found below: https://db.cngb.org/cnsa/; CNP0002205.

## References

[B1] Abel H. J. Larson D. E. Larson D. E. Regier A. A. Chiang C. Das I. (2020). Mapping and Characterization of Structural Variation in 17,795 Human Genomes. Nature 583, 83–89. 10.1038/s41586-020-2371-0 32460305 PMC7547914

[B2] Bertelsen B. Nazaryan-Petersen L. Sun W. Mehrjouy M. M. Xie G. Chen W. (2016). A Germline Chromothripsis Event Stably Segregating in 11 Individuals through Three Generations. Genet. Med. 18, 494–500. 10.1038/gim.2015.112 26312826

[B3] Carvalho C. M. B. Vasanth S. Shinawi M. Russell C. Ramocki M. B. Brown C. W. (2014). Dosage Changes of a Segment at 17p13.1 lead to Intellectual Disability and Microcephaly as a Result of Complex Genetic Interaction of Multiple Genes. Am. J. Hum. Genet. 95, 565–578. 10.1016/j.ajhg.2014.10.006 25439725 PMC4225592

[B4] Chau M. H. K. Cao Y. Kwok Y. K. Y. Chan S. Chan Y. M. Wang H. (2019). Characteristics and Mode of Inheritance of Pathogenic Copy Number Variants in Prenatal Diagnosis. Am. J. Obstet. Gynecol. 221, 493.e1–493.e11. 10.1016/j.ajog.2019.06.007 31207233

[B5] Chau M. H. K. Wang H. Lai Y. Zhang Y. Xu F. Tang Y. (2020). Low-Pass Genome Sequencing: a Validated Method in Clinical Cytogenetics. Hum. Genet. 139, 1403–1415. 10.1007/s00439-020-02185-9 32451733

[B6] Collins R. L. Brand H. Redin C. E. Hanscom C. Antolik C. Stone M. R. (2017). Defining the Diverse Spectrum of Inversions, Complex Structural Variation, and Chromothripsis in the Morbid Human Genome. Genome Biol. 18, 36. 10.1186/s13059-017-1158-6 28260531 PMC5338099

[B7] de Pagter M. S. Van Roosmalen M. J. Baas A. F. Renkens I. Duran K. J. Van Binsbergen E. (2015). Chromothripsis in Healthy Individuals Affects Multiple Protein-Coding Genes and Can Result in Severe Congenital Abnormalities in Offspring. Am. J. Hum. Genet. 96, 651–656. 10.1016/j.ajhg.2015.02.005 25799107 PMC4385185

[B8] Del Gaudio D. Shinawi M. Astbury C. Tayeh M. K. Deak K. L. Raca G. (2020). Diagnostic Testing for Uniparental Disomy: a Points to Consider Statement from the American College of Medical Genetics and Genomics (ACMG). Genet. Med. 22, 1133–1141. 10.1038/s41436-020-0782-9 32296163

[B9] D'haene E. Vergult S. (2021). Interpreting the Impact of Noncoding Structural Variation in Neurodevelopmental Disorders. Genet. Med. 23, 34–46. 10.1038/s41436-020-00974-1 32973355 PMC7790743

[B10] Dong Z. Jiang L. Yang C. Hu H. Wang X. Chen H. (2014). A Robust Approach for Blind Detection of Balanced Chromosomal Rearrangements with Whole-Genome Low-Coverage Sequencing. Hum. Mutat. 35, 625–636. 10.1002/humu.22541 24610732

[B11] Dong Z. Zhang J. Hu P. Chen H. Xu J. Tian Q. (2016). Low-Pass Whole-Genome Sequencing in Clinical Cytogenetics: a Validated Approach. Genet. Med. 18, 940–948. 10.1038/gim.2015.199 26820068

[B12] Dong Z. Yan J. Xu F. Yuan J. Jiang H. Wang H. (2019a). Genome Sequencing Explores Complexity of Chromosomal Abnormalities in Recurrent Miscarriage. Am. J. Hum. Genet. 105, 1102–1111. 10.1016/j.ajhg.2019.10.003 31679651 PMC6904795

[B13] Dong Z. Zhao X. Li Q. Yang Z. Xi Y. Alexeev A. (2019b). Development of Coupling Controlled Polymerizations by Adapter-Ligation in Mate-Pair Sequencing for Detection of Various Genomic Variants in One Single Assay. DNA Res. 26, 313–325. 10.1093/dnares/dsz011 31173071 PMC6704401

[B14] Dong Z. Chau M. H. K. Zhang Y. Yang Z. Shi M. Wah Y. M. (2021). Low-pass Genome Sequencing-Based Detection of Absence of Heterozygosity: Validation in Clinical Cytogenetics. Genet. Med. 23, 1225–1233. 10.1038/s41436-021-01128-7 33772221 PMC8522200

[B15] Fan Y.-S. Ouyang X. Peng J. Sacharow S. Tekin M. Barbouth D. (2013). Frequent Detection of Parental Consanguinity in Children with Developmental Disorders by a Combined CGH and SNP Microarray. Mol. Cytogenet. 6, 38. 10.1186/1755-8166-6-38 24053112 PMC3853444

[B16] Ho S. S. Urban A. E. Mills R. E. (2020). Structural Variation in the Sequencing Era. Nat. Rev. Genet. 21, 171–189. 10.1038/s41576-019-0180-9 31729472 PMC7402362

[B17] Hoppman N. Rumilla K. Lauer E. Kearney H. Thorland E. (2018). Patterns of Homozygosity in Patients with Uniparental Disomy: Detection Rate and Suggested Reporting Thresholds for SNP Microarrays. Genet. Med. 20, 1522–1527. 10.1038/gim.2018.24 29565418

[B18] Kaminsky E. B. Kaul V. Paschall J. Church D. M. Bunke B. Kunig D. (2011). An Evidence-Based Approach to Establish the Functional and Clinical Significance of Copy Number Variants in Intellectual and Developmental Disabilities. Genet. Med. 13, 777–784. 10.1097/gim.0b013e31822c79f9 21844811 PMC3661946

[B19] Lelieveld S. H. Reijnders M. R. F. Pfundt R. Yntema H. G. Kamsteeg E.-J. De Vries P. (2016). Meta-analysis of 2,104 Trios Provides Support for 10 New Genes for Intellectual Disability. Nat. Neurosci. 19, 1194–1196. 10.1038/nn.4352 27479843

[B20] Li H. Durbin R. (2009). Fast and Accurate Short Read Alignment with Burrows-Wheeler Transform. Bioinformatics 25, 1754–1760. 10.1093/bioinformatics/btp324 19451168 PMC2705234

[B34] Manickam K. Mcclain M. R. Demmer L. A. Biswas S. Kearney H. M. Malinowski J. (2021). Exome and Genome Sequencing for Pediatric Patients With Congenital Anomalies or Intellectual Disability: An Evidence-Based Clinical Guideline of the American College of Medical Genetics and Genomics (ACMG). Genet Med. 10.1038/s41436-021-01242-6 34211152

[B21] Miller D. T. Adam M. P. Aradhya S. Biesecker L. G. Brothman A. R. Carter N. P. (2010). Consensus Statement: Chromosomal Microarray Is a First-Tier Clinical Diagnostic Test for Individuals with Developmental Disabilities or Congenital Anomalies. Am. J. Hum. Genet. 86, 749–764. 10.1016/j.ajhg.2010.04.006 20466091 PMC2869000

[B22] Mooneyham K. A. Holden K. R. Cathey S. Dwivedi A. Dupont B. R. Lyons M. J. (2014). Neurodevelopmental Delays and Macrocephaly in 17p13.1 Microduplication Syndrome. Am. J. Med. Genet. 164, 2887–2891. 10.1002/ajmg.a.36708 25123844

[B23] Moutton S. Bruel A.-L. Assoum M. Chevarin M. Sarrazin E. Goizet C. (2018). Truncating Variants of the DLG4 Gene Are Responsible for Intellectual Disability with Marfanoid Features. Clin. Genet. 93, 1172–1178. 10.1111/cge.13243 29460436

[B24] Newman S. Hermetz K. E. Weckselblatt B. Rudd M. K. (2015). Next-generation Sequencing of Duplication CNVs Reveals that Most Are Tandem and Some Create Fusion Genes at Breakpoints. Am. J. Hum. Genet. 96, 208–220. 10.1016/j.ajhg.2014.12.017 25640679 PMC4320257

[B25] Palumbo P. Palumbo O. Leone M. P. Stallone R. Palladino T. Zelante L. (2015). Maternal Uniparental Isodisomy (iUPD) of Chromosome 4 in a Subject with Mild Intellectual Disability and Speech Delay. Am. J. Med. Genet. 167, 2219–2222. 10.1002/ajmg.a.37142 25994769

[B26] Pócza T. Grolmusz V. K. Papp J. Butz H. Patócs A. Bozsik A. (2021). Germline Structural Variations in Cancer Predisposition Genes. Front. Genet. 12, 634217. 10.3389/fgene.2021.634217 33936164 PMC8081352

[B27] Srivastava S. Love-Nichols J. A. Dies K. A. Ledbetter D. H. Martin C. L. Chung W. K. (2019). Meta-analysis and Multidisciplinary Consensus Statement: Exome Sequencing Is a First-Tier Clinical Diagnostic Test for Individuals with Neurodevelopmental Disorders. Genet. Med. 21, 2413–2421. 10.1038/s41436-019-0554-6 31182824 PMC6831729

[B28] Talkowski M. E. Rosenfeld J. A. Blumenthal I. Pillalamarri V. Chiang C. Heilbut A. (2012). Sequencing Chromosomal Abnormalities Reveals Neurodevelopmental Loci that Confer Risk across Diagnostic Boundaries. Cell 149, 525–537. 10.1016/j.cell.2012.03.028 22521361 PMC3340505

[B29] Wang H. Dong Z. Zhang R. Chau M. H. K. Yang Z. Tsang K. Y. C. (2020). Low-pass Genome Sequencing versus Chromosomal Microarray Analysis: Implementation in Prenatal Diagnosis. Genet. Med. 22, 500–510. 10.1038/s41436-019-0634-7 31447483 PMC7042067

[B30] Werling D. M. Brand H. An J.-Y. Stone M. R. Zhu L. Glessner J. T. (2018). An Analytical Framework for Whole-Genome Sequence Association Studies and its Implications for Autism Spectrum Disorder. Nat. Genet. 50, 727–736. 10.1038/s41588-018-0107-y 29700473 PMC5961723

[B31] Yu T. Li J. Li N. Liu R. Ding Y. Chang G. (2016). Obesity and Developmental Delay in a Patient with Uniparental Disomy of Chromosome 2. Int. J. Obes. 40, 1935–1941. 10.1038/ijo.2016.160 27654142

[B32] Yuan B. Wang L. Liu P. Shaw C. Dai H. Cooper L. (2020). CNVs Cause Autosomal Recessive Genetic Diseases with or without Involvement of SNV/indels. Genet. Med. 22, 1633–1641. 10.1038/s41436-020-0864-8 32576985 PMC8445517

[B33] Yuan H. Shangguan S. Li Z. Luo J. Su J. Yao R. (2021). CNV Profiles of Chinese Pediatric Patients with Developmental Disorders. Genet. Med. 23, 669–678. 10.1038/s41436-020-01048-y 33402738

