# Supplementary material for: Investigation of Chromosomal Structural Abnormalities in Patients With Undiagnosed Neurodevelopmental Disorders
Source: Front Genet. 2022 Apr 14;13:803088. doi: 10.3389/fgene.2022.803088 (PMC9046776; doi:10.3389/fgene.2022.803088)
Supplement: Supplementary file 1 [file DataSheet1.docx]

**Supplementary Materials**

Supp Table S1. The clinical characteristics of this cohort.

|  | male | female | No. |
| --- | --- | --- | --- |
| Neurodevelopmental conditions with or without comorbid of features | 62 | 25 | 87 |
| With other system abnormalities | 9 | 4 | 13 |
| Total | 71 | 29 | 100 |

Supp Table S2. Additional AOH findings in three cases of this cohort

| case ID | clinical indications | AOH regions (>5Mb) |
| --- | --- | --- |
| 41 | Global developmental delay | seq[GRCh37]aoh(6)(q14.3q15)chr6:g.85100000_90300000hmz;  aoh(6)(q25.1q25.2)chr6:g.149200000_154300000hmz; |
| 44 | Delay | seq[GRCh37]aoh(4)(p15.33p15.31)chr4:g.14800000_19900000hmz;  aoh(16)(q23.1q23.2)chr16:g.74500000_80300000hmz;  aoh(19)(p13.2p13.12)chr19:g.9000000_14700000hmz; |
| 76 | Autism and delay | multiple chromosomes with AOH(214 Mb) |

Supp Table S3. Primer sequence of qPCR validation for three small deletions

| Case ID | Primer Pair | Forward Primer (5'->3') | Reverse Primer(5'->3') |
| --- | --- | --- | --- |
| Case15 | Primer pair-1 | TTCCTCAGTCTTACCCCAAATCA | TTGACGGTAGTTGGTTTTATGGG |
| Case15 | Primer pair-2 | AACAATGGGTAAGAAAGAGCACC | ATTGGCGTCATACAACCACTTAC |
| Case21 | Primer pair-1 | ATGAAGTGAAATGTCCCCGAATC | GATGTGTGTTTCAGTAGATGGCA |
| Case21 | Primer pair-2 | TTCCATCATCTTTGTGAGCCATC | GGTTAGGTGAGAGGCTACAGTAA |
| Case42 | Primer pair-1 | AAAATATGGACTCTCATGCCCAC | AAAGGAACAGTGTTATGCTTGGT |
| Case42 | Primer pair-2 | AGCCTCAACCATGTATCCAGTAC | CTCCAACACAGACTTATTCACCA |

Supp Figure S1. Schematic representations of the complex rearrangement on 17p13.1 detected in the case 19 (upper) and three breakpoints X, Y ,Z all validated by Sanger sequencing (below).


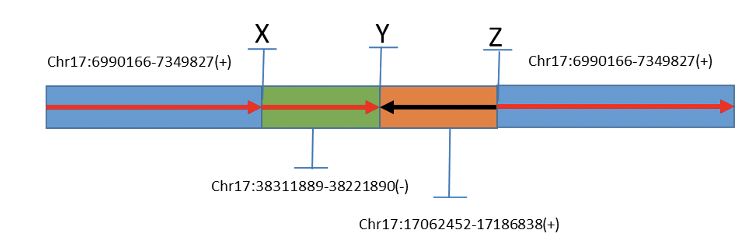


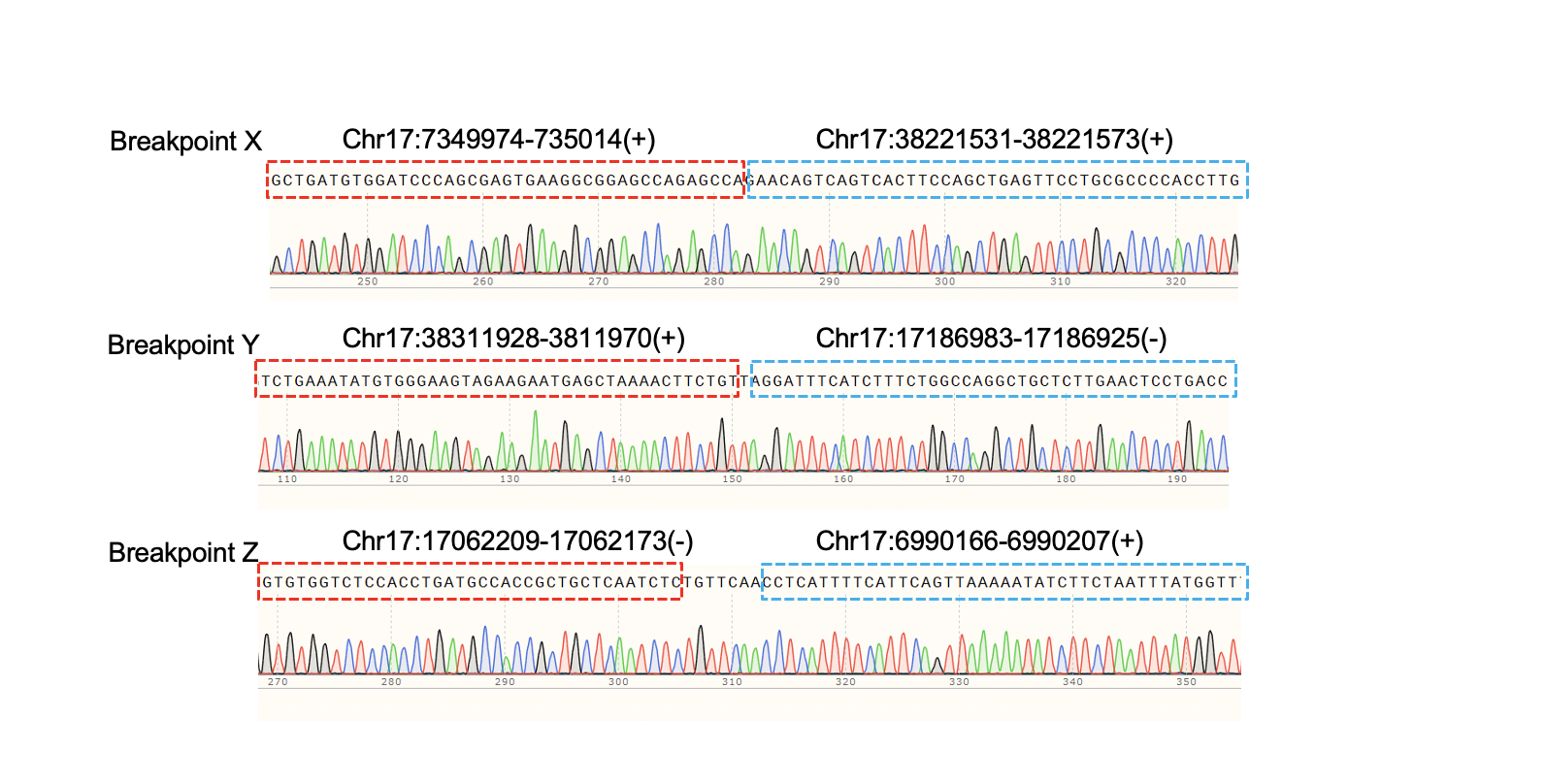


Supp Figure S2. qPCR validation of additional three deletions detected by mate-pair low-pass sequencing.


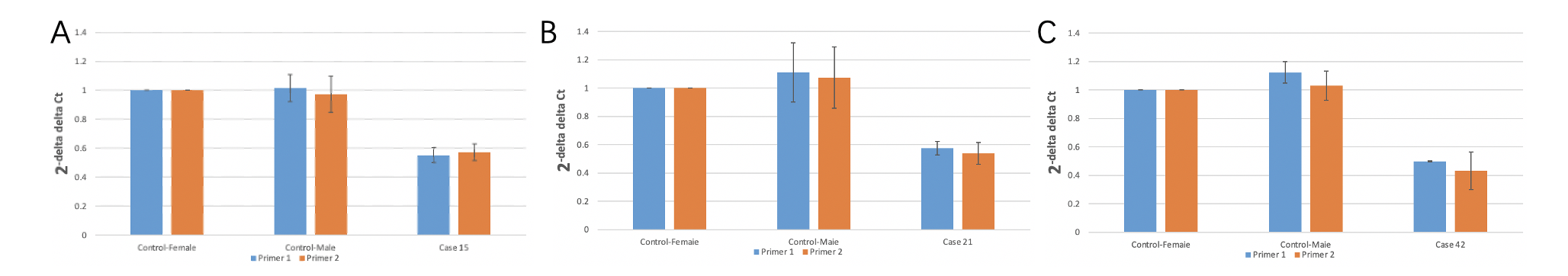


Bar charts show the results of qPCR suggested the deletions were true in case15, case 21 and case 42
